# Awareness and Treatment Decisions on Tooth Wear among Jordanian Dentists and Prosthodontists: A Cross-Sectional Survey Study

**DOI:** 10.1155/2020/8861266

**Published:** 2020-11-24

**Authors:** Samiha Sartawi, Nesreen A. Salim, Duaa Taim

**Affiliations:** ^1^Department of Prosthodontics, School of Dentistry, University of Jordan, Amman, Jordan; ^2^Jordan University Hospital, Amman, Jordan

## Abstract

**Objectives:**

To assess the awareness, knowledge, and treatment decisions by dentists in Jordan regarding tooth wear.

**Materials and Methods:**

A questionnaire was disseminated to a random sample of 200 general dentists and 100 prosthodontists working in the Ministry of Health, academia, private practices, and military services. Chi square and independent *t*-tests were performed for statistical analysis.

**Results:**

Hundred and seventy-nine dentists and prosthodontists responded (59.7% response rate), of which 71.5% was females. 83.8% of the dentists reported they see patients with tooth wear. 61.5% registered wear lesions in the patient file, and 68.2% reported they find a probable cause of tooth wear. 87.2% of the dentists reported that bruxism is the most common cause in Jordan. 63.3% dentists treated their patients. 46.4% reported they “always” record a dietary history. 77.7% did not think that tooth wear is linked to caries. Low confidence levels were demonstrated among general practitioners in diagnosing and treating tooth wear. Regarding treatment decisions, most dentists decided to restore worn teeth with composite and to construct a night guard. Minimally affected anterior teeth were mostly treated with fluoride. Restoration of posterior worn teeth with overlay was suggested by one-third of the dentists.

**Conclusion:**

The dentists and prosthodontists in Jordan are aware of tooth wear. However, examination and documentation were given a little priority by general dentists. On the other hand, there was an agreement among the dentists and prosthodontists on applying the minimally invasive approach. *Clinical Significance*. It is challenging for dentists to make the best treatment decision for tooth wear especially as no standard treatment is available. Therefore, this study investigated the awareness and treatment decisions of a sample of dentists and prosthodontists in Jordan.

## 1. Introduction

Tooth wear is a multifactorial irreversible loss of dental hard tissues by a combination of mechanical and chemical processes not involving bacterial acids [1]. Tooth wear involves four individual processes which are attrition, abrasion, erosion, and abfraction. As defined by the ORCA and the Cariology Research Group of the IADR, tooth wear is the cumulative surface loss of mineralized tooth substance due to physical or chemophysical processes (dental erosion, attrition, and abrasion) [2]. Tooth wear or tooth surface loss (TSL) is not a new phenomenon; however, with the change in life style, eating habits, and socioeconomic factors, it is becoming more prevalent in both developed and developing countries [3, 4]. Generally, it has been reported that age-related tooth surface loss ranged from 15 *μ*m to 29 *μ*m per year depending on the tooth [5]. But for some, particularly those with reflux-related symptoms, the wear exceeded 100 *μ*m in a period of six months [5].

The percentage of adults presenting with severe tooth wear has increased from 3% at the age of 20 to 17% at the age of 70, which means there is a significant relationship with increasing age [6]. A more recent study showed that in children and adolescents, the estimated prevalence of erosive wear in permanent teeth is 30% in Europe [5]. This percentage conforms to the systematic review and meta-regression analysis that was carried out in 2015 [7]. Another study showed that the percentage of exposed dentine was 23.4% in the general population of Germany [8]. Furthermore, a study investigating tooth wear in five-year-old children in Jakarta, Indonesia, revealed that tooth wear occurred in 23% of the participants [9]. However, in Jordan, dentine exposure was found in 51% of 15- to 16-year-old school children [10], indicating that tooth wear among Jordanian children has highly exceeded the prevalence rates in young patients worldwide. Another study of a group of Jordanian adults found that out of the 306 patients studied, 80% showed clinical signs of tooth surface loss. In the previous study, the mandibular arch was more affected with wear than the maxillary arch, and the anterior segments were more affected with wear compared to the posterior segments [11].

The treatment of dentitions affected by tooth wear should be based on the following biological aims: (1) preservation of remaining tooth structure, (2) improvement of esthetics, and (3) longevity of restorations [12]. Different management protocols to treat localized and generalized tooth wear are presented in case reports with little information on tooth wear aetiology. Obviously, there is no globally accepted treatment method for such cases. However, minimal invasive dentistry has overcome the traditional full mouth rehabilitation approach to manage worn dentitions with more focus on protecting the remaining tooth tissue [12]. Direct adhesive restorative materials are currently accepted among clinicians in the UK for restoring tooth wear because this approach is cost-effective and can address both functional and aesthetic needs of patients an early stage of tooth wear and minimizes further damage of the teeth [13].

While most of the literature focus is on management of erosive tooth wear [2], Mehta et al. 2012 published a four-part series that address the current concepts on the management of tooth wear in general [14–17]. The authors considered accurate diagnosis to be the cornerstone of a successful treatment. The etiological factors should be identified, and a preventive management should be constructed accordingly, and passive monitoring should be carried out to assess the progression of localized or generalized tooth wear. Any further treatment is preferred to be fully reversible and adjustable [15]. In the management of localized tooth wear, reversible and additive techniques should be implemented as a first approach in the active restorative intervention followed by conventionally retained indirect restorations as a second option [16]. In 2017, a European Consensus Statement on Management Guidelines was published, and it aimed at differentiating pathological tooth wear and severe tooth wear, in addition to outlining a proper management that takes into consideration not only the severity and effects of the tooth wear but also the patient's wishes. It concluded that a conservative, minimally invasive approach is only to be taken when the preventive measures for further tooth wear fail, since the restorative phase is seemingly perpetual due to the nature of tooth wear. They also emphasized on the importance of risk assessment as the primary key for a successful management. To sum up, the European consensus advises a meticulous risk assessment of the patient, seeking alternative forms of management such as prevention and monitoring and keeping in mind the effects of further wear that could lead to failure of restorations and prostheses [5]. A recent series by Hemmings et al. in 2018 have recommended guidelines for the management of tooth wear according to the British Society of Restorative Dentistry (BSRD), including fixed and removable treatment options and prevention [18–20]. However, it is still apparent that there is no clear recipe for the management of tooth wear. One can speculate that this variability between clinicians is most likely due to the lack of consensus in the literature, the absence of universal diagnostic criteria, and clear-cut definitions to help differentiate between the various tooth wear conditions.

As the variability continues, levels of dentists' knowledge have also fluctuated notably between countries around the world. For example, it was found that only half of Yemeni dentists and dental students had deep knowledge about etiological factors, diagnosis, and prevention of dental erosion [21]. In addition, a Brazilian sample of dentists, dental students, and patients demonstrated lack of understanding and communication on dental erosion [22]. On the other hand, Mulic et al. studied Norwegian and Icelandic dentists, both of which were up-to-date regarding diagnosis and treatment, but certain aspects such as dietary and salivary analyses were not given the priority needed [23, 24]. The levels of knowledge regarding tooth wear among Jordanian dentists have not been measured before. Therefore, the aim of this study was to gain knowledge about the awareness and treatment decisions the dentists and prosthodontists in Jordan offer to different tooth wear processes by a questionnaire survey.

## 2. Materials and Methods

The study protocol was granted an ethical approval by the Deanship of Academic Research at the institution where the study was conducted. Participation was voluntary, and no compensation was given. A computer-generated random table was used to ensure anonymity among participants.

A self-constructed questionnaire was disseminated electronically to a total of three hundred general dentists (GP) and prosthodontists (PD) (GP, *N* = 200 and PD, *N* = 100) through social media (WhatsApp, Facebook, messenger, and e-mail). The sample size was calculated using the “Sample Size Calculator” available freely online (https://www.abs.gov.au/websitedbs/d3310114.nsf/home/sample+size+calculator). The questionnaire targeted a random sample working in the private sector, Ministry of Health, academia, and military health services in March 2020. A database of all dentists and prosthodontists in Jordan was requested from the Jordanian Dental Association. The database included 4555 registered GPs and 163 PDs (accessed in March 2020).

Each dentist received an invitation letter, an information sheet, and a consent form that explained the aims of the study and assured the dentists that the data collected will be completely anonymous. The questionnaire was derived from questionnaires focused on erosive tooth wear from previous studies [23, 24], while in the present study, the questionnaire was modified to assess the knowledge and management strategies of tooth wear in general. The new version of the questionnaire was piloted and validated across a group of 10 dentists before the initiation of the study. The questionnaire contained twenty-three questions which were distributed across three sections. The first section of the questionnaire gathered demographic information about the participants (place of residence and work, sex, age, speciality, last degree obtained and from which country, years of experience, and the field of practice).

The second section targeted dentists' awareness and knowledge of tooth wear. The questions enquired information on whether the participants see patients with tooth wear and if they register the condition in the patient's file. Also, the dentists were asked whether they find a probable reason for tooth wear, record a dietary history, and find a connection between tooth wear and caries activity among patients presented with tooth wear. At the end of this section, dentists were asked to grade their confidence levels regarding the diagnosis and treatment of tooth wear on a visual analogue scale (VAS) ranked from unconfident to confident on a 10-point scale [25].

The third section involved three clinical patient cases where the dentists were asked to decide on the treatment for every case. A very brief patient history besides coloured clinical photographs of the labial, palatal, and occlusal surfaces of upper and lower teeth with different tooth wear aetiologies and severities were provided. The three clinical cases were collected from previous studies [23, 24] and from the author's own clinical practice.

The questions are described below.

**Figure 1:**
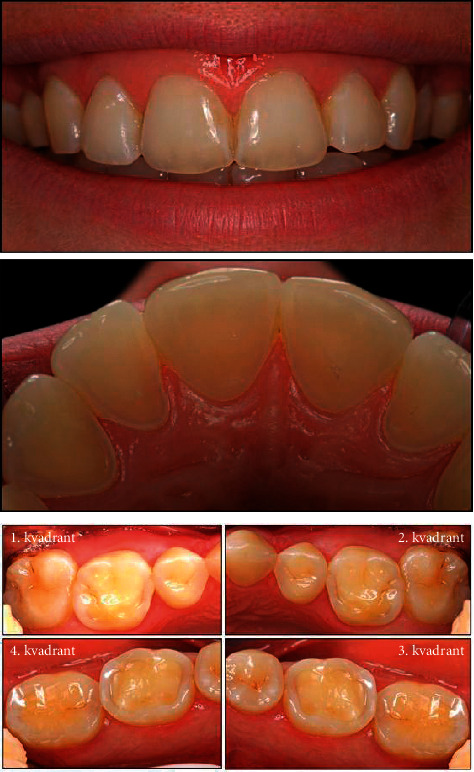
Clinical case one (Mulic et al., 2012 and Mulic et al., 2018).

Case one: the patient is a 28-year-old woman, Figure 1.

  Question one: how would you treat the maxillary front region (you can make more than one choice)? (1) No treatment. (2) Treat locally with fluoride solution/bonding agent. (3) Restore with composite. (4) RCT. (5) Restore with a crown. (6) Construct a night guard.
  Question two: how would you treat the maxillary and mandibular first molars (you can make more than one choice)? (1) No treatment. (2) Treat locally with fluoride solution/bonding agent. (3) Restore with composite. (4) Restore with overlay/onlay. (5) RCT. (6) Restore with a crown. (7) Construct a night guard.

**Figure 2:**
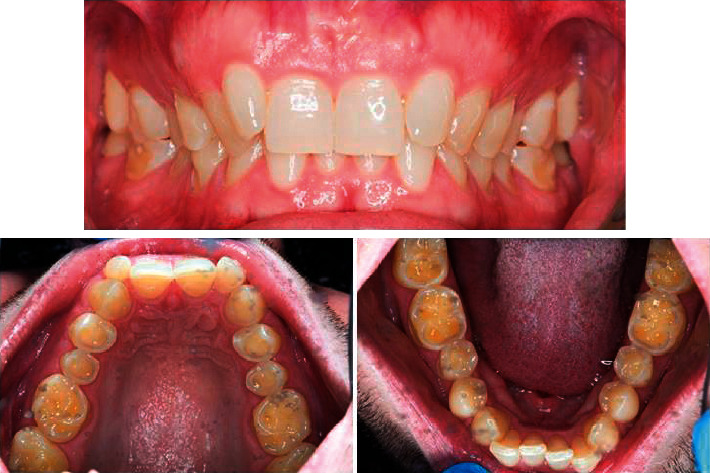
Clinical case two.

Case two: the patient is a 30-year-old male with a history of GORD, Figure 2.

  Question one: how would you treat the maxillary premolar region (you can make more than one choice)?
  Question two: how would you treat the mandibular first and second molars (you can make more than one choice)? The options for question one and two were similar to case one treatment options.

**Figure 3:**
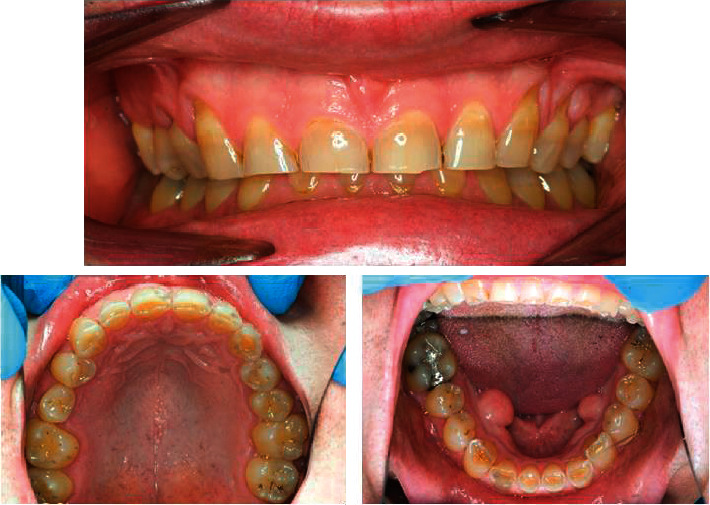
Clinical case three.

Case three: a 50-year-old male. Fit and healthy but clenches on his anterior teeth unconsciously, Figure 3.

Only one question asked about the treatment options of maxillary and mandibular anterior region with the treatment options similar to question one in case one.

  Statistics: the data were processed using SPSS version 18.0.3 (Statistical Package for the Social Sciences; SPSS Inc., Chicago, Ill., USA), and statistical evaluation was carried out by means of descriptive statistics. Chi square was used to measure the association between different variables. The independent *t*-test with a 95% confidence interval was used to measure the difference in confidence levels between dentists and prosthodontists regarding diagnosis and treatment of the clinical cases.

## 3. Results

### 3.1. Demographics

Hundred and eighty four GPs and PDs out of a total of three hundred clinicians completed the questionnaire, which accounted to a total response rate of 59.7% (*n* = 121 GPs, 67.7% and *n* = 60 PDs, 32.4%). The dentists ranged in age from 23 to above 50 years and consisted of 71.5% females and 28.5% males. The distribution of dentists according to age, gender, speciality, qualification, work place, and years of experience is presented in Table 1.

### 3.2. Awareness and Knowledge of Tooth Wear

Frequencies and percentages (%) of GPs and PDs' answers to questions measuring awareness and knowledge of tooth wear is presented in Table 2. Hundred and fifty dentists (83.8%) reported that they often see patients with tooth wear in their practice, while 14.5% reported always, and 1.7% reported they never seen tooth wear patients. Most dentists (*n* = 110, 61.5%) registered tooth wear lesions in their patient's file and 38% (*n* = 68) did not. For those who did not register tooth wear, 30.7% (*n* = 55) reported that tooth wear was not the chief complaint of the patient, 6.1% (*n* = 11) were not sure how to register, and a minority of the dentists (*n* = 8, 4.5%) found tooth wear difficult to diagnose.

Hundred and twenty-two dentists (68.2%) reported that they mostly find a probable cause of tooth wear, 29.1% occasionally, and only 2.8% reported that they seldom find a probable cause. Chi square statistics showed that there were no correlation between the frequency the dentists seeing patients with tooth wear in their practices nor finding a probable cause with age, speciality, years of experience, and work place (*P* > 0.05). With regards to registration in the patient file, PDs tended to register tooth wear more than GPs as shown by chi square statistics (*X*^*2*^ = 5.4, *P* < 0.05).

The most common causes of tooth wear in Jordan as given by the dentists were bruxism (87.2%), consumption of acidic foods and drinks (24.6%), and gastroesophageal reflux (8.4%) followed by rampant caries (4.5%) as a more uncommon cause. Regarding bruxism, chi square analysis detected an association between the variables years of experience (*X*^*2*^(3) = 11.7, *P* < 0.05) and age (*X*^*2*^(3) = 12.5, *P* < 0.05) with the selection of this cause.

Hundred and twelve dentists (63.3%) treated their patients presented with tooth wear themselves, while 25.7% referred the patients to speciality/university clinic and 10.6% referred them to another dentist. Chi square analysis revealed a strong relationship between the decision to refer or treat tooth wear with speciality (*X*^*2*^(2) = 36.9, *P* < 0.05), years of experience (*X*^*2*^(2) = 27.8, *P* < 0.05), age (*X*^*2*^(6) = 35.4, *P* < 0.05), and gender (*X*^*2*^(2) = 7.7, *P* < 0.05).

Eighty-three dentists (46.4%) reported that they “always” recorded a dietary history for patients with tooth wear, while 26.3% (*n* = 47) reported that they “often” recorded a dietary history, 21.8% (*n* = 39) only “occasionally,” and 5.6% (*n* = 10) reported that they “never” recorded dietary history. There was a strong association between taking a dietary history and speciality (*X*^*2*^(3) = 19.5, *P* < 0.05) with 69% of PDs always recording a dietary history compared to 35.5% of GPs.

Regarding the connection between tooth wear and caries activity in patients, most of the dentists (*n* = 139, 77.7%) did not think that patients with tooth wear had more caries than those without. However, 12.3% (*n* = 22) of the dentists thought there is more caries activity among tooth wear patients, and 10.1% (*n* = 18) of the dentists did not know there is a connection. There was a strong relationship between the beliefs in the connection between tooth wear and caries, speciality (*X*^*2*^(2) = 7.2, *P* < 0.05), and age (*X*^*2*^(6) = 13.2, *P* < 0.05) as demonstrated by chi square statistics.

**Figure 4:**
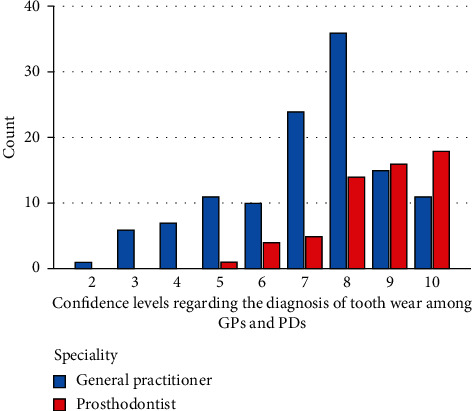
The frequency of confidence levels of GPs and PDs regarding the diagnosis of tooth wear on a 10-point VAS scale.

**Figure 5:**
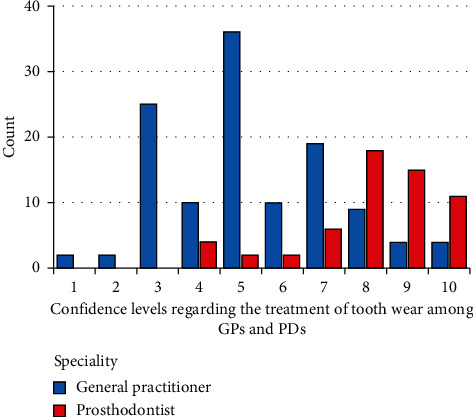
The frequency of confidence levels of GPs and PDs regarding the treatment of tooth wear on a 10-point VAS scale.

The frequency of confidence levels of the GPs and PDs regarding the diagnosis of tooth wear on a VAS scale is represented in Figure 4. The frequency of confidence levels of the GPs and PDs regarding the treatment of tooth wear on a VAS scale is represented in Figure 5. The independent sample *t*-test revealed a significant difference between dentists and prosthodontists with regards to confidence levels for both diagnosis and treatment of tooth wear that prosthodontists were more confident (*P*=0.0001). The mean scores for diagnosis were 7.14 and 8.67 out of 10 for GPs and PDs, respectively, while the mean scores for the treatment were 5.31 and 8.09 for GPs and PDs, respectively (1 being not confident at all and 10 being very confident).

### 3.3. Strategies of Management

For patient case one, the frequency of treatment decisions with the number of dentists responding to each treatment option is presented in Table 3.

Regarding the treatment decision, treatment of anterior maxillary teeth by local fluoride administration was reported by half of the dentists (*n* = 102, 57%), while 36.9% decided to restore with composite followed by 34.6% who decided not to treat, and a minority of the dentists decided to do RCT (*n* = 2, 1.1%). Chi square analysis revealed a strong relationship between the decision to restore with composite and a kind of association with the decision to construct a night guard and speciality (*X*^*2*^(1) = 12.2, *P*=0.0001 and *X*^*2*^(1) = 4.46, *P*=0.03), respectively.

**Figure 6:**
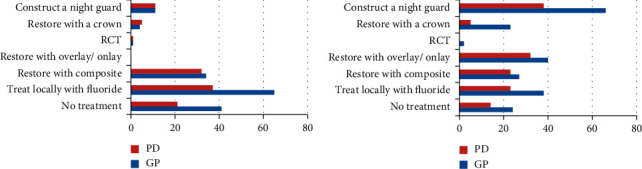
Frequencies of treatment decisions among GPs (general practitioners) and PDs (prosthodontists).

With regards to the treatment of posterior teeth, half of the dentists (58.1%) decided to construct a night guard, 40.2% to restore with an overlay, 34.1% to treat locally with fluoride, and 27.9% to restore with composite. Statistical analysis demonstrated a strong association between the decision to restore with composite or with an overly and being a GP or a PD (*X*^*2*^(1) = 5, *P*=0.025 and *X*^*2*^(1) = 5.75, *P*=0.018), respectively.  Figure 6 depicts the frequencies of treatment decisions among GPs and PDs for case one.

**Figure 7:**
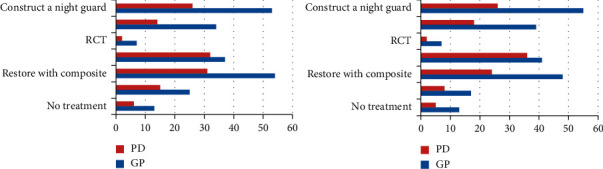
Frequencies of treatment decisions among GPs (general practitioners) and PDs (prosthodontists) regarding case two.

For patient case two, the frequencies of treatment decisions with the number of dentists responding to each treatment option is presented in Table 4. Dentists' decisions were fairly close regarding the treatment of maxillary premolars. 47.5% of the dentists decided to restore the premolars with composite, 44.1% to construct a night guard, and 38.5% to restore with overlay. Regarding the treatment of mandibular first molars for the clinical case two, 44.7% of the dentists decided to construct a night guard, 43% to restore with overlay, 40.2% to restore with composite, and 31.3% to construct a crown. Chi square analysis revealed a strong relationship between the decision to restore with overlay for maxillary premolars, mandibular first molars, and speciality (*X*^*2*^(1) = 9.9, *P*=0.002 and *X*^*2*^(1) = 13.4, *P*=0.0001), respectively.  Figure 7 depicts the frequencies of treatment decisions among GPs and PDs for this case.

**Figure 8:**
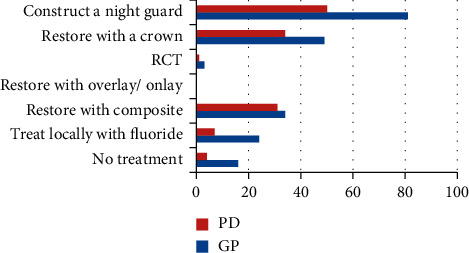
Frequencies of treatment decisions among GPs (general practitioners) and PDs (prosthodontists) regarding case three.

Table 5 presents the frequencies of treatment decisions with the number of dentists responding to each treatment option regarding patient case three.  Figure 8 depicts the frequencies of treatment decisions among GPs and PDs for this case. The majority of dentists 73.2% decided to construct a night guard to treat the maxillary front teeth, 46.4% to construct a crown, and 36.3% to restore with composite. The decision to perform RCT was selected by a minority of the dentists (2.2%). There was a relationship between the decision to restore the maxillary teeth with composite and to construct a crown and speciality as demonstrated by chi square statistics (*X*^*2*^(1) = 4.14, *P*=0.04 and *X*^*2*^(1) = 5.17, *P*=0.02).

## 4. Discussion

A change in the paradigm in management of dentitions affected by tooth wear has been emphasized in the late years that severe tooth wear should be treated with minimally invasive approaches wherever possible [5]. Therefore, this study aimed at evaluating the awareness, knowledge, and treatment strategies of general practitioners and prosthodontists in Jordan regarding this clinical condition. The present study is the first to analyse and compare the knowledge and treatment decisions of tooth wear between GPs and PDs in Jordan. A survey of a random sample working in different work sectors in the country was conducted, and the response rate of 59.7% reported in this study was in accordance with similar studies reported in the literature [23, 24]. The results of this study demonstrated a general agreement among dentists with regards to treatment. However, some inconsistencies were detected among the two study groups related to the awareness and knowledge of examination and assessment of tooth wear cases.

The majority of the dentists (83.3%) reported they see patients with tooth wear in their practises. This means that tooth wear is common in Jordan, although there are no epidemiological studies on the prevalence rate of the condition up-to-date except one study carried out on school children [10] and another study on a sample of 306 adults [11]. Most dentists (61.5%) reported they register tooth wear in the patient's file and 38% did not. 30.7% of the dentists who did not register stated that tooth wear was not the chief complaint of the patient. This is in accordance with the nature of tooth wear which is asymptomatic, and patients usually present to dental practices for urgent interventions or routine dental procedures [11]. Similarly, 68.2% of the dentists reported to find a probable cause of tooth wear which is similar to previous studies [23, 24]. It was encouraging that dentists were aware of tooth wear and can find a probable cause regardless of their speciality, age, years of experience, and work place. However, prosthodontists were more likely to register the condition in the patient file. One may speculate that prosthodontists tend to spend more time for extended examination and documentation, while general practitioners consider this to be time-consuming [23].

The majority of dentists (87.2%) reported that the most important cause of tooth wear in Jordan is bruxism followed by the consumption of acidic foods and drinks (24.6%). The analysis of the results demonstrated a strong association between the selection of bruxism as the main cause of tooth wear with years of experience and age. Younger dentists who have less years of experience tend to identify bruxism less than elder dentists. One can expect that fresh dentists were more likely to have learnt about the multifactorial aetiology of tooth wear during their undergraduate education, so they did not select one cause only. Another possible explanation for these results is the absence of large scale demographic studies on tooth wear in Jordan. Although all tooth wear processes interact together to produce tooth surface loss, the role of attrition (bruxism) is thought to be overestimated in explaining severe wear processes, indeed no strong evidence to prove that attrition is an active etiological factor [26].

Regarding the referral for treatment of tooth wear, most of the dentists (62.6%) reported that they do the treatment of their own patients with tooth wear, and 36.3% referred the patients to a university clinic or to another dentist. This was in accordance with the referral pattern in similar studies that focused on the treatment of dental erosion [24]. However, there was a strong association between the tendency to refer and speciality, age, years of experience, and gender in contrast to previous studies [23, 24, 27]. Not surprising that prosthodontists were more competent to treat the patients themselves. This result is in accordance with a similar survey among prosthodontists in the UK [27]. On the other hand, younger dentists who had fewer years of experience and female dentists tend to refer rather than treat tooth wear by themselves. Interestingly, a pilot study of 124 referred tooth wear cases revealed that attrition was the primary etiological cause of tooth wear in 51% of referrals, and of the total referred cases, only 8% required a specialist's treatment [28].

Dietary history is extremely important in order to identify the possible etiological factors in patients presented with tooth wear [18]. In the present study, only 46.4% of the dentists reported they always record a dietary history which was similar to the results reported among Norwegian and Iceland dentists [23, 24]. Although recording a dietary history was a low priority for dentists, the prosthodontists were more likely to spend time on dietary history, which is consistent with previous results in the current study. This is very important as the effective prevention of the progression of tooth wear, especially erosion, often requires an individualized dietary advice.

The results demonstrated a low confidence in diagnosing and treating tooth wear cases among general practitioners compared to prosthodontists. This is similar to the results obtained from Leeds dentists where the mean score for restoring dentitions with complex wear was 4.65 out of 10 [25]. This finding might be worrying because our survey also demonstrated that 62.6% of the dentists, of which 47.9% of the GPs, reported that they treat the patients themselves. A prospective survey of secondary care tooth wear referrals in Leeds demonstrated that 59% of the referred patients were from deprived areas [28]. This variable was not measured in our survey as more focus was on the confidence to treat tooth wear or refer if unconfident. Another study concluded that dentists had difficulty in diagnosing and managing erosive tooth wear especially in early stages when compared to caries [29].

One important purpose of the current study was to identify the treatment strategies for tooth wear performed by the dentists in Jordan. This was illustrated with three clinical cases (Figures 1–3). The patient case one (Figure 1) was identical with the one included in previous studies, which was a combination of mild enamel and severe dentine lesions [23, 24]. The clinical case two and three were obtained from the author's log of clinical cases. The patient case two presented a patient with more severe erosive lesions on maxillary premolars and maxillary and mandibular molars than that seen in the patient case one. The clinical case three illustrated a patient with severe dentine lesions on maxillary and mandibular six anterior teeth as a result of unconscious clenching.

For the patient case one, about half of the dentists decided to treat the anterior teeth locally with fluoride, while one-third of the dentists decided to restore with composite and one-third to do nothing. Treatment with topical fluoride might be beneficial in preventing the progression of enamel erosion in the early stages of the wear process. In vitro treatment of enamel with topical fluoride prior to acidic challenge was found to inhibit initial erosion by increasing enamel hardness, inhibition of softening [30], and increasing abrasion resistance of enamel against mechanical insults [31]. Similarly, a quarter of the dentists decided to construct overlay for the posterior teeth, one-third decided to treat the posterior teeth with topical fluoride administration, and one-third to restore with composite. These results were in accordance with the decisions made by the Nordic and Iceland dentists, except for the overlay decision which was not included in those studies [23, 24]. This was also in accordance with the current trend toward a minimally invasive approach when treating erosive lesions. However, in the current survey, no specification for the teeth requiring management was performed, and instead, the decision was made for a whole quadrant. This might result in some confusion among the dentists during clinical decision-making as the upper central incisors presented with mild enamel erosion, while the upper lateral incisors and first molars showed more severe dentine involvement which might necessitate operative intervention. Interestingly, about half of the dentists decided to construct a night guard to manage the erosive tooth wear on the posterior teeth. On the other hand, prosthodontists were more likely to perform operative procedures including composite restorations and overlays to treat tooth wear. This might be explained by that the prosthodontists' focus was on the teeth exhibiting dentine exposure rather than the sound ones in the quadrant. In a similar survey, direct composites were most commonly chosen by the UK prosthodontists to treat palatal erosive lesions not involving incisal edges [27].

Regarding case two, which was more severe than case one, the frequencies of treatment decisions were distributed on restoring with composite, construction of night guard, and overlays, respectively, for the maxillary premolars and night guard, overlays, and composite, respectively, for the mandibular molars. This trend might be difficult to interpret, especially when the molars are affected; the dentists tended to construct a night guard regardless of the aetiology of tooth wear, as demonstrated by the results of case one. Occlusal splints have been commonly used by dentists to prevent tooth wear caused by bruxism and heavy loading of the teeth [32] unlike the reasons they were prescribed for cases one and two in the current study. On the other hand, the prosthodontists were more likely to restore the maxillary premolars and molars with overlays as demonstrated by the statistical analysis and may be because more emphasis on the minimal invasive approach is being incorporated in postgraduate studies and dental conferences. Although the adhesive restorative options such as overlays and onlays are being used widely nowadays, no information is available for the treatment of tooth wear applying these newer techniques [33].

For case three, which was for an individual with localised tooth wear on the upper and lower anterior teeth due to clenching, most of the dentists decided to construct a night guard followed by crowning the affected teeth and then to restore with composite, respectively. The prosthodontists were more likely to restore the anterior teeth with composite (53.4%) and crowns (58.6%) compared to general practitioners. May be, they prefer to start with a reversible less destructive treatment option before proceeding with a more invasive clinical decision to construct crowns. Jordanian prosthodontists were similar to their UK colleagues in the treatment of short clinical crowns and wear on the lower incisors using resin-based materials [27]. Composite restorations have been used to treat localized anterior tooth wear and have been shown to be effective over a 10-year period with some maintenance [34].

The three clinical cases provided in the current study were of the localised tooth wear category where only a few number of teeth are affected by wear processes. However, the degree of tooth surface loss varied between the cases with an attempt to monitor the treatment strategies the dentists in Jordan could perform for each case. A brief history was given to guide the dentists through the aetiology of tooth wear. No information was given to the dentists regarding the vertical dimension of occlusion which may affect their treatment decision. As recommended by The European Consensus in 2017, the management of severe tooth wear should be minimally invasive wherever possible, in order to keep as many as possible future restorative options [5]. This is of course after addressing the aetiology and the progression rate of tooth wear. However, there is not an ideal material to treat tooth wear, and there is a compromise between aesthetic considerations and durability [19] and no strong evidence to suggest that one material is better than another [35]. Whether direct or indirect materials may be used to restore severely worn teeth, direct composite resins were recommended to treat less severe tooth wear cases according to Anterior Clinical Erosion classification (ACE) [35]. It is still debatable whether starting with a less invasive technique is preferable rather than a highly aggressive but more resistant one eventually [35]. It is obvious that the prosthodontists and dentists in Jordan were aware that there was lack of evidence on the management of tooth wear; so, the treatment decisions were less invasive to protect the affected dentition with either local fluoride administration or the construction of night guard. However, the decision to construct a night guard was overprescribed for cases one and two because the aetiology was erosion from extrinsic acids for the former and intrinsic acids for later. One may speculate that the night guard was selected in adjunct to other restorative decisions and not as the sole treatment option.

Further studies are needed to update the prevalence rate of tooth wear in Jordan on a large population scale. In addition, this work might be expanded to explore the influence of many variables on dental wear, such as general health conditions, e.g., diabetes type 2 [36], oral acid environment, life style, and dietary intake of acids [37]. Tooth brushing is also one of the variables in relation to the wear of natural teeth [38] and dental materials [39, 40]. Nonetheless, the use of different restorative materials for the treatment of tooth wear needs to be further explored both in vitro and in vivo. It was stated that wear of teeth and composite materials can be due to bruxism and/or gastroesophageal reflux disease [41]. Indirect restorative materials on contrary may induce wear of natural teeth [42]. In addition, sleeping disorders including orofacial pain, oral dryness, GERD, and sleep bruxism have been linked to tooth wear and have a synergetic effect to accelerate the wear process [43]. On the other hand, drugs could be related to tooth wear either directly or indirectly by affecting the amount and/or flow of saliva or influencing the acidity of the oral cavity [44]. For example, the drugs used for the treatment of asthma [45] and antiretroviral therapy in HIV patients [46] were linked to tooth wear. Therefore, future reports are needed to complete the results of the present study evaluating also these important variables.

A limitation of the current study was inability to send individual reminders to participants over the social media which might have an influence on the sample size and return rate. In addition, the clinical scenarios that were provided did not take into account the patient's aesthetic or functional requirements nor the patient's financial status. These important factors always affect the clinical decision-making process. Another limitation of the study was in the questionnaire design that multiple responses were accepted for the treatment decisions. It might be more valid to select and rank the decisions according to their priority or hierarchical order if more than one option was to be selected.

## 5. Conclusion

This study suggests that the dentists and prosthodontists in Jordan are aware of tooth wear processes. However, little priority was given by the general dentists to examination and documentation in the patient file. Low confidence levels in diagnosing and treating tooth wear among general practitioners are worrying, although there was an agreement with prosthodontists on applying the minimally invasive approach when treating tooth wear cases. Prevention of further tooth damage was achieved by providing a night guard to all tooth wear cases. It is important to have in mind that when the restorative treatment of worn dentition is indicated, resin composite should be the first material of choice. However, the need for conventional treatments remains in some cases, but minimum intervention should always be considered first.

## Figures and Tables

**Table 1:** The distribution of dentists according to age, gender, speciality, qualification, work place, and years of experience.

		Dentists	
		*N*	%
Age	23–29	66	36.9
	30–39	66	36.9
	40–49	30	16.2
	>50	16	8.9

Gender	Male	51	28.5
	Female	128	71.5

Speciality	General practitioner	120	67.6
	Prosthodontist	58	32.4

Qualification	BDS/DDS	111	62
	Jordanian board	9	5
	Master's degree	41	22.9
	PhD	18	10.1

Work place	Private clinic	97	54.2
	Ministry of health	39	21.8
	Academia	40	22.3
	Military services	18	10.1

Years of experience	1–5 years	74	41.3
	6–10 years	30	16.8
	11–20 years	47	26.3
	>20 years	28	15.6

**Table 2:** Frequencies and percentages (%) of GPs and PDs' answers to questions measuring awareness and knowledge of tooth wear.

Question	Dentists	
	No.	%
How often do you see patients with tooth wear in your practice		
Always	26	14.5
Often	150	83.8
Never	3	1.7

Do you register tooth wear in the patient file		
Yes	110	61.8
No	68	38.2

If no, why do not you register tooth wear		
I am not sure how to register	11	6.1
I find tooth wear difficult to diagnose	8	4.5
It is not the chief complaint of the patient	55	30.7

Do you usually find a probable cause for tooth wear		
Mostly not	5	2.8
Occasionally	52	29.1
Mostly yes	122	68.2

What do you think is the most common cause of tooth wear in Jordan		
Bruxism	156	87.2
Consumption of acidic foods and drinks	44	24.6
Rampant caries	8	4.5
Gastroesophageal reflux	15	8.4

What do you do if you have a patient with tooth wear requiring treatment		
I refer him/her to a speciality/university clinic	46	26
I refer him/her to another dentist	19	10.7
I treat him/her myself	112	63.3

Do you take a dietary history of patients who present with tooth wear		
Always	83	46.4
Never	10	5.6
Occasionally	39	21.8
Often	47	26.3

Do you think that people with tooth wear have more caries		
I do not know	18	10.1
No	139	77.7
Yes	22	12.3

**Table 3:** The frequency of treatment decisions and (*n*) the number of GPs and PDs responding to each treatment option for clinical case one.

Treatment decision	How would you treat the maxillary front region? *n* (%), GP (%), PD (%)			How would you treat the maxillary and mandibular first molars? *n* (%), GP (%), PD (%)		
No treatment	62 (34.6)	42 (34.7)	20 (34.5)	38 (21.2)	24 (19.8)	14 (24.1)
Treat locally with fluoride	102 (57)	66 (54.5)	36 (62.1)	61 (34.1)	40 (33.1)	23 (38.3)
Restore with composite	66 (36.9)	34 (28.1)	32 (55.2)^*∗*^	50 (27.9)	27 (22.3)	23 (39.7)^*∗*^
Restore with overlay/onlay	—	—	—	72 (40.2)	41 (33.9)	31 (53.4)^*∗*^
RCT	2 (1.1)	1 (0.8)	1 (1.7)	2 (1.1)	2 (1.7)	0 (0)
Restore with a crown	9 (5)	4 (3.3)	5 (8.6)	28 (15.6)	23 (19.0)	5 (8.6)
Construct a night guard	22 (12.3)	11 (9.1)	11 (19)^*∗*^	104 (58.1)	68 (56.2)	38 (62.1)

^*∗*^Significant difference between PGs and PDs regarding the treatment decision; *P* < 0.05.

**Table 4:** The frequency of treatment decisions and (*n*) the number of GPs and PDs responding to each treatment option on clinical case two.

Treatment decision	How would you treat the maxillary premolar region? *n (*%), GP (%), PD (%)			How would you treat the mandibular first and second molars? *n (*%), GP (%), PD(%)		
No treatment	19 (10.6)	13 (10.7)	6 (10.3)	18 (10.1)	13 (10.7)	5 (8.6)
Treat locally with fluoride	40 (22.3)	25 (20.7)	15 (25.9)	25 (14)	19 (15.7)	6 (10.3)
Restore with composite	85 (47.5)	54 (44)	31 (53.4)	72 (40.2)	48 (39.7)	24 (41.4)
Restore with overlay/onlay	69 (38.5)	37 (30.6)	32 (55.2)^*∗*^	77 (43)	41 (33.9)	36 (62.1)^*∗*^
RCT	8 (6.6)	7 (5.9)	1 (1.7)	9 (5)	7 (5.8)	2 (3.4)
Restore with a crown	48 (26.8)	35 (28.9)	13 (22.4)	56 (31.3)	39 (32.2)	17 (29.3)
Construct a night guard	79 (44.1)	55 (44.6)	25 (43.1)	80 (44.7)	56 (46.3)	24 (41.4)

^*∗*^Significant difference between PGs and PDs regarding the treatment decision, *P* < 0.05.

**Table 5:** The frequency of dentists' treatment decisions and (*n*) the number of GPs and PDs responding to each treatment option on clinical case three.

Treatment decision	How would you treat the maxillary front region?		
	*n (*%)	GP (%)	PD (%)
No treatment	20 (11.2)	16 (13.2)	4 (6.9)
Treat locally with fluoride	31 (17.3)	25 (20.7)	6 (10.3)
Restore with composite	65 (36.3)	34 (28.1)	31 (53.4)^*∗*^
Restore with overlay/onlay	—	—	—
RCT	4 (2.2)	3 (2.5)	1 (1.7)
Restore with a crown	83 (46.4)	49 (40.5)	34 (58.6)^*∗*^
Construct a night guard	131 (73.2)	83 (68.6)	48 (82.8)

^*∗*^Significant difference between PGs an PDs regarding the treatment decision, *P* < 0.05.

## Data Availability

The data (original data are in the SPSS version 24.0 (Statistical Package for the Social Sciences; SPSS Inc., Chicago, Ill., USA)) and the questionnaire used to support the findings of this study are available from the corresponding author upon request.
